# What Next After MBSR/MBCT? An Open Trial of an 8-Week Follow-on Program Exploring Mindfulness of Feeling Tone (*vedanā*)

**DOI:** 10.1007/s12671-022-01929-0

**Published:** 2022-07-07

**Authors:** J. Mark G. Williams, Ruth Baer, Martine Batchelor, Rebecca S. Crane, Chris Cullen, Kath De Wilde, Melanie J. V. Fennell, Linda Kantor, Janine Kirby, S. Helen Ma, Emma Medlicott, Barbara Gerber, Mandy Johnson, Ee-Lin Ong, John W. Peacock, Danny Penman, Andy Phee, Lucy Radley, Matthew Watkin, Laura Taylor

**Affiliations:** 1 Department of Psychiatry, University of Oxford, Warneford Hospital, Oxford, OX3 7JX UK; 2 Aquitaine, France; 3 Centre for Mindfulness Research and Practice, School of Psychology, Bangor University, College Road, Bangor, LL57 2DG UK; 4 Institute of Mindfulness, South Africa, Suite 10 7th Floor Auto Atlantic B, Foreshore, Cape Town, 8018 Western Cape South Africa; 5 Graduate School of Business, University of Cape Town, Rondebosch, 7701 South Africa; 6 Center for Mindfulness, Hong Kong, 121 Des Voeux Road Central, Room 1701, Far East Consortium Building Central, Hong Kong, Hong Kong; 7 Oxford Mindfulness Centre, Department of Psychiatry, Warneford Hospital, Oxford, OX3 7JX UK; 8 Nature & Nurture Sparks, Christchurch, New Zealand; 9 Bodhi College, 4 Dartside, Totnes, TQ9 5HL Devon UK; 10 Bristol, UK; 11 MindfulN16, London, UK

**Keywords:** Mindfulness-based program, Feeling tone, *Vedanā*, Harm, Side effects

## Abstract

**Objectives:**

The effectiveness of mindfulness-based programs (MBPs) has been established in many randomized controlled trials. However, effect sizes are often modest, and there remains ample scope to improve their effectiveness. One approach to this challenge is to offer a “follow-on” course to people who have completed an MBP and are interested in further skill development. We developed and tested a new 8-week course for this purpose based on awareness of feeling tone (*vedanā*), an understudied aspect of mindfulness in many current MBPs, incorporating new developments in neuroscience and trauma sensitivity. We examined its effectiveness and the frequency and severity of unpleasant experience and harm.

**Methods:**

In an open trial, 83 participants, 78 of whom had previously taken part in an MBP (majority MBSR or MBCT), completed the program in nine groups. Participants completed questionnaires before and after and gave qualitative written feedback at completion.

**Results:**

Participants reported significantly reduced depression (*d* = 0.56), stress (*d* = 0.36), and anxiety (*d* = 0.53) and increased well-being (*d* = 0.54) and mindfulness (*d* = 0.65) with 38% meeting criteria for reliable change on anxiety and depression. As expected, about three-quarters of participants reported some unpleasant experiences associated with mindfulness practice during the course, but none reported harm. Five participants showed “reliable deterioration” (an increase) in either depression or anxiety, but four of these five also gave anonymous qualitative feedback describing benefits of the course.

**Conclusions:**

Findings support the added value of a follow-on course based on the exploration of feeling tone for participants who have a range of previous mindfulness experience.

**Supplementary Information:**

The online version contains supplementary material available at 10.1007/s12671-022-01929-0.

The effectiveness of mindfulness-based programs (MBPs) has been established in many randomized controlled trials. Goldberg et al. (2021) examined 44 meta-analyses of RCTs involving MBPs that included 336 trials and a total of 30,483 participants. They found a consistent pattern of effectiveness across many different populations (Goldberg et al., 2021). The conclusion of such meta-analyses is that MBPs consistently show superiority to passive controls across most populations, problems, interventions, comparisons, and outcomes. There is also evidence that MBPs are as effective or more effective than active controls for some conditions, with the most robust evidence for addictions, pain, and depression. However, although these meta-analyses show that MBPs justify their place alongside other evidence-based treatments, the effect sizes emerging from studies vary, and there remains ample scope to improve their effectiveness.

To date, there have been three main responses to this challenge. First, to offer booster sessions or follow-on series, to sustain, broaden, and deepen the themes taught in the original MBP (e.g., *Mindful Living Practice Groups* offered by the Center for Mindfulness in San Diego, or the *Taking it Further* course offered by the Oxford Mindfulness Centre), though to our knowledge, the effectiveness of these has not yet been evaluated. A second strategy is to develop programs that are more tailored to specific diagnoses, such as health anxiety (McManus et al., 2012), obsessive compulsive disorder (Didonna et al., 2019; Selchen et al., 2018), and cancer (Carlson & Garland, 2005). A third strategy is to offer programs which extend mindfulness into cognate areas, such as Mindfulness-based Compassionate Living (Van den Brink & Koster, 2015) and Interpersonal Mindfulness (Kramer et al., 2008) . Each of these strategies shows promise (Bartels-Velthuis et al., 2016; Schuling et al., 2021; Z. Kramer, 2015). But these approaches leave unanswered whether participants could sustain and deepen their practice by exploring an aspect of mindfulness that is claimed to be foundational for the development of mindfulness and is taught in retreat, but not explicitly addressed in MBSR and MBCT: awareness of feeling tone (*vedanā*).

According to Early Buddhist psychological theory as found in the texts of the Pali *Suttas*, *Abhidhamma*, and commentaries, *vedanā* is the second foundation of mindfulness, and an essential element in the emergence of any moment of conscious experience (Bodhi, 2005; Nānamoli & Bodhi, 1995; Walshe, 1995). It is one of the five essential factors acting together with embodiment and environment in any moment: contact, feeling tone, perception, attention, and intention to act (Batchelor, 2019). Feeling tone is also one of the five physical and mental “aggregates” involved in the onset and maintenance of craving (Thiṭṭila, 1995), and one of the twelve co-dependent links that explain the way that conscious experience is patterned, unless the chain is interrupted by the development of mindfulness at the point when contact and feeling tone arise and are noted (Anālayo, 2018).

In over two and a half millennia of Buddhist scholarship and practice, there has been some divergence of interpretation concerning the nature of *vedanā*. It has been translated as body sensations, sensations, body feelings, feelings (with the danger of being confused with emotions), hedonic tone, and feeling tone. The program evaluated in the current study uses the term *feeling tone* (Bodhi, 2005), defining it as the immediate, automatic, experiential sense that any contact with the external or internal world is pleasant or unpleasant, or neither. According to this interpretation, *vedanā* is neither a body sensation, nor an emotion, nor a cognitive judgment of the experience with which contact has been made, but rather an instant, automatic, wordless felt-sense of where an experience falls on the hedonic spectrum between extremely pleasant to extremely unpleasant. So, Bodhi (2012) stated that *vedanā* is “the bare affective quality of an experience, which may be either pleasant, painful or neutral” (p. 80), and Batchelor (2018) defined *vedanā* as “the pleasant, unpleasant and neutral tonality of experience that arises upon contact through the six senses with one’s outer or inner environment” (p. 57). By focussing on *vedanā* as “feeling tone,” the investigative scope includes mental phenomena as well as somatic sensations.

No matter how *vedanā* has been interpreted throughout its history, the aim of training has been to break the link between *vedanā* and reactive tendencies that arise dependent upon it. This is seen particularly clearly in the Buddhist theory of Dependent Co-arising (*paṭiccasmuppāa*) that offers a diagnostic as to how experience is patterned in automatic and reactive ways. At the heart of this patterning lies the connection between *vedanā* and the reaction: *taṇhā* or “craving”—a desire to prolong the pleasant and avoid the unpleasant. Unpleasant feeling tones, for example, trigger underlying tendencies to aversion that lie not with the object but within the individual. Feeling tone, together with either aversion or desire, is key to understanding the development of emotional reactivity and allows for some kind of contemplative or meditative intervention that leads to clear seeing of the vital difference between the feeling tone and emergent reactive pattern (Webster, 2005). The specific cultivation of awareness of feeling tone thus promises to give practitioners valuable early warning signs of impending impulses, thoughts, and emotional reactions, and hence more choice about what perspective and action are likely to be helpful (Weber, 2018).

Despite its importance, Peacock and Batchelor (2018) pointed out that in both Buddhist-based teaching contexts and in much of the secular mindfulness movement, far less attention is given to *vedanā* than to the other foundations of mindfulness. ”Great attention, for example, is focused on the body, mind and hindrances, yet *vedanā* is skirted over fairly rapidly” (p. 1). Is this true of Mindfulness-Based Stress Reduction (MBSR; Kabat-Zinn, 2013) and Mindfulness-Based Cognitive Therapy (MBCT, Segal et al., 2013)? To be sure, there is extensive emphasis on mindfulness of body sensations in MBSR and MBCT (body scan, mindful movement practices, mindfulness of the breath, and, in MBCT, the practice of turning towards difficulty by sensing where the difficulty affects the body). This means that, in these programs, there is already emphasis on cultivating mindful awareness of *vedanā* in the sense of “body sensations” and equanimity towards them. Indeed, the emphasis on developing sustained attentiveness to body sensations in MBSR/MBCT has been suggested to be a main driver of therapeutic change (Kerr, et al., 2013; Williams, 2010). Kerr et al. (2013) suggested that the body scan, the first formal practice in both MBSR and MBCT, is a key to understanding other critical processes that mediate improved mental and physical health, as it promotes just the sort of flexibility of attentional filtering that they show is characteristic of experienced practitioners in their brain imaging (MEG) studies.

But if *vedanā* is defined specifically as the *feeling tone* of any experience, then it remains under-explored in MBSR/MBCT despite its hypothesized pivotal role in the exacerbation and maintenance of emotional distress. Although *vedanā—*as thus defined*—*is proving helpful in the addiction field (Brewer, et al., 2013), it remains largely implicit in MBSR/MBCT. It is explored most explicitly using the “pleasant/unpleasant experiences calendar” home practice (and the enquiry that follows this assignment) in MBSR/MBCT and, in MBCT in “recognizing aversion.” Unlike in some retreat contexts, there are no formal practices that focus exclusively on *vedanā*, so participants are not systematically taught to cultivate awareness of it.

The need to clarify how feeling tone arises and what effects it has on mental and physical functioning has been given additional impetus by recent cognitive neuroscience, particularly the field of predictive processing and embodied cognition. Research in predictive processing shows that the brain is constantly active in making “top down” predictions on the basis of our remote and recent past, and that much of our conscious experience of the internal and external world (including interoception) arises from these predictions rather than direct perception in the present (Clark, 2015; Farb, et al., 2015; Manjaly & Iglesias, 2020). Research on embodied cognition shows that our mental life is dependent on computing the action that our bodies need to take to operate in the world, so that understanding the world and other people involves a series of simulated or imagined actions (Barsalou, 2008; Gjelsvik et al., 2018). The feeling tone generated by sense data from the outer and inner worlds, and from the predictive mental models simulated from them, determines the direction and urgency of action (Barrett, 2018; Damasio & Carvalho, 2013;). As the body prepares and “budgets” for such actions, interoceptive changes contribute to the feeling tone of each moment (Barrett and Simmonds, 2015). Barrett (2006) concluded that an affect system, with valence at its core, constitutes the most basic building block of emotional life—its core process.

However, there are risks in exploring the core process that underlies emotion, as inviting participants to become ever more aware of the very “tipping point” moments can create distress. Though such awareness is central to the “exposure” aspect of mindfulness practice, it can overwhelm the participant (Treleaven, 2018). Several studies have pointed to the potential harmful effects of meditation in general, and the invitation to be open to all experience in particular, arising from the reactivation of traumatic memories (Baer et al., 2019; Lindahl et al., 2017). Although several randomized trials have shown that adverse effects, when reported, are no more common in MBPs than in control groups (Hirshberg et al., 2020; Wong et al., 2018), questions have been raised about the definition of harm in these studies. Most studies have failed to distinguish difficult emotions that are experienced as a predictable aspect of any therapeutic procedure. They may be transitory phenomena affording opportunities to learn to work skilfully with difficulty or exacerbation of symptoms that are overwhelming, where the participant feels unable to cope using the methods taught (Baer, et al., 2019). Baer et al. (2019) found that in those studies that assess harm, there are increases in symptoms (or the appearance of new symptoms) in 0 to 11% of participants, a frequency of deterioration that suggests an important need to take account of harm. Treleaven (2018) draws upon a model to explain the “windows of tolerance” for such difficult experiences (Siegel, 1999) and suggests a number of trauma-sensitive strategies that mindfulness teachers and participants can use to mitigate the effects.

The aim of the current study was to investigate (a) whether a new 8-week program that focuses on feeling tone would be acceptable and safe for participants already familiar with mindfulness practice through MBSR/MBCT (or programs related to them); (b) whether and to what extent it would decrease stress, anxiety, and depression and increase mindfulness and well-being; (c) whether and to what extent it would cause harm; and (d) whether any beneficial or harmful outcomes differed depending on the extent of prior experience of mindfulness practice.

## Method

### Participants

**Fig. 1:**
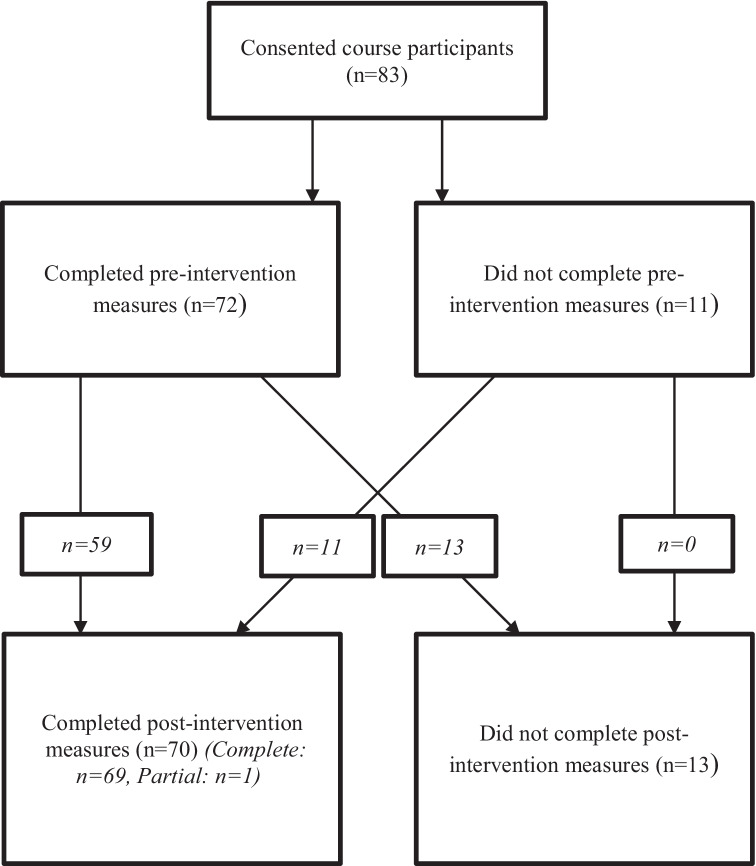
Consort diagram

The study used an opportunity sample of participants who had previously taken part in 8-week mindfulness courses at mindfulness teaching centers in the centers in the Institute of Mindfulness South Africa, in London, and in Christchurch, New Zealand. Eighty-three participants provided data, across nine different training groups; 69% of participants were female (*n* = 50). Participants were aged 24 to 77 (M = 50.09, SD = 11.71). Eighty-nine percent of participants (*n* = 64) were employed, 7% (*n* = 5) were unemployed, and 4% (*n* = 3) were retired (Fig. 1).

Although the program was designed as a follow-on course for those who have previously attended some form of mindfulness training, we did not specify how much prior mindfulness experience was needed. Of the 72 participants who completed the baseline measures, 93.1% of participants had previous experience of practicing mindfulness (*n* = 67) and 6.9% had no experience of practicing mindfulness (*n* = 5). Of those 67 participants who had experience practicing mindfulness, 3% had been practicing for 0–3 months (*n* = 2), 9% had been practicing for 3–12 months (*n* = 6), 48% had been practicing for 1–5 years (*n* = 32), and 40% had been practicing for over 5 years (*n* = 27).

The majority of the participants had gained mindfulness meditation experience through completing an evidence-based MBP (MBSR or MBCT or a program closely based on these). Seventy-two participants described their mindfulness training using a multi-response option (where more than one response could be selected), 13% said “I know very little but would like to know more” (*n* = 9), 3% said “I have done a mindfulness course through a book” (*n* = 2), 83% said “I have done a mindfulness course with an instructor in a class” (*n* = 60), 51% said “I have attended a retreat” (*n* = 37), and 39% responded “other” (*n* = 28). A minority of participants had not completed an 8-week course, and this was permitted as one aim of the study was to check whether the extent of experience affected outcome, so including a few participants with less experience allowed us to sample a larger range.

Participants completed questionnaires prior to and after completion of the course, and also gave qualitative written feedback on the course, week by week and at completion. Seventy-two participants provided baseline data and 59 participants provided data at both pre- and post-intervention. There were no significant differences between those who provided complete data and those who completed only the pre-intervention questions (*n* = 13) on the baseline measures. Similarly, there were no significant differences between those who provided complete data and those who completed only the post-intervention questions (*n* = 11) on the outcome measures (see Supplementary Information 2).

### Procedure

The course was developed in Oxford by JMGW, based on teachings given in 1- and 2-day retreats at international mindfulness meetings (alongside CC, HM, and RSC), 4-day retreats co-taught with HM between 2014 and 2019 for participants on the Foundation Course at the Hong Kong Center for Mindfulness, and input from scholars and teacher-trainers in contemporary Buddhism and MBCT (MB, JWP, CC, and MJVF).

A 6-week version using a central core of feeling tone practices was piloted in Oxford, North Wales, and South Africa. The course was further developed with two additional sessions added, one prior to the six-session core to teach participants how to handle difficult experiences, based on trauma-sensitive elements recommended by Treleaven (2018), and a final session for reviewing the course, integrating mindfulness into everyday life, and planning future practice. The final course (Williams & Penman, 2023) is outlined in Table 1.

**Table 1:** Overview of the 8-week program

Week	Session theme	Summary of contents
1	Finding your ground	Exploring different strategies and a range of anchors (as well as the breath) for when the mind/body is restless or overwhelmed by difficulty or trauma
2	The Pause: befriending and gathering the scattered mind	Exploring ways of befriending and gathering the mind when lost in rumination and worry, by pausing to register any self-judgment of mind-wandering and cultivate gratitude and understanding of what the mind is trying to do
3	Discovering the feel-of-things	Registering the “feeling tone” (pleasant, unpleasant or neither) of whatever arises, moment by moment, starting with sounds, body sensations, or thoughts in meditation practice and in everyday life
4	Restoring balance	Learning to *allow* feeling tones as a way of staying balanced. Discovering the freedom that comes from seeing tonality as natural, and from giving permission to like what is pleasant and not to like what is unpleasant
5	Feeling-tone at the fringes of consciousness	Registering the feeling tone moment by moment to see more clearly subtle changes that arise from ongoing activity in mind and body at the fringe of consciousness, and noticing when the mind is gearing up for unnecessary action
6	Approaching difficult emotions	Focusing on body sensations that occur with emotions, sensing their feeling tone (as in Week 3) allowing them (as in Week 4) and letting go of a need for immediate action (as in Week 5) as way of cultivating insight and kindness
7	Reclaiming your life	Exploring the intimate connection between feeling tone, mood and activity, recognizing reactivity to feeling tone, using awareness of feeling tone as a wise guide towards skilful action
8	Deepening mindfulness, cultivating wisdom	Supporting intentions to be mindful, including awareness and responsiveness to feeling tone, less trapped in the “driven mode” brought on by reactivity to feeling tone, and considering a “year-of-practice” guide to help this

Senior mindfulness teachers in New Zealand (E-LO), South Africa (BG, MJ, LK, JK, MW, and the UK (AP) recruited participants (with prior experience of mindfulness) and asked if they would be willing to take part in evaluation of the new program. It was explained that the course had been designed for those who are already familiar with mindfulness and offered an opportunity to deepen and extend their meditation practice. All participants completed a consent form. The study was approved by the Oxford University Ethics Committee (ER67367/RE001) and data analysis was conducted in Oxford (KW, LR, EM, LT) independently of the mindfulness teachers. A total of nine classes were run.

Self-report data were collected at two time points, pre-course (T0) and post-course (T1). Participants were sent a link via email to the pre- and post-questionnaires (hosted on Qualtrics) by their mindfulness teacher. Participants were given a unique participant identification number to maintain confidentiality throughout the study. Participants were made aware that their participation was voluntary and would not have any bearing on their course participation. Participants were not compensated for their time or participation. They signed a digital informed consent form and were given the contact details of the research team for any questions they might have. If participants responded above the clinical cutoff for any of the symptom questionnaires (Patient Health Questionnaire, Kroenke et al., 2001; or the General Anxiety Disorder, Spitzer et al., 2006), they were given information about who they could contact for support (including their mindfulness instructor), in accordance with our ethics application (Oxford University Ethics Committee (ER67367/RE001).

### Measures

Participants gave socio-demographic data and completed pre- and post-course measures to assess any changes in depression and anxiety that could then be compared with their responses to post-course unpleasant experience and harm questions (mirroring the assessments of harm in Baer et al., 2021).

Depressive symptomology was measured using the Patient Health Questionnaire (PHQ-9; Kroenke et al., 2001). The PHQ-9 is a 9-item measure which assesses severity of depression in the preceding 2 weeks (e.g., “Feeling down, depressed or hopeless”). Items are rated on a 4-point Likert-type scale (from 0 = “Not at all” to 3 = “Nearly every day”). Scores can range from 0 to 27. Total scores of 20 and higher are considered clinically significant. Internal reliability was very good in the current sample (T0: Cronbach’s *α* = 0.79, McDonald’s *ω* = 0.79; T1: Cronbach’s *α* = 0.85, McDonald’s *ω* = 0.85).

Anxiety symptoms were measured using the General Anxiety Disorder Scale (GAD-7; Spitzer et al., 2006), which measures anxiety symptoms over the past 2 weeks (e.g., feeling nervous, anxious or on edge). Items are rated on a 4-point Likert-type scale (from 0 = “Not at all” to 3 = “Nearly every day”). Scores can range from 0 to 21. Total scores of 15 and higher are considered clinically significant. Internal reliability in the current sample was good (T0: Cronbach’s *α* = 0.90, McDonald’s *ω* = 0.90; T1: Cronbach’s *α* = 0.89, McDonald’s *ω* = 0.89).

The Perceived Stress Scale (PSS; Cohen & Janicki-Deverts, 2012) was used to assess symptoms of stress over the past month (e.g., “In the last month, how often have you felt that things were going your way?”). Ten items are rated on a 5-point Likert-type scale (from 0 = “Never” to 4 = “Very often”). The scale had good internal reliability (T0: Cronbach’s *α* = 0.85, McDonald’s *ω* = 0.84; T1: Cronbach’s *α* = 0.86, McDonald’s *ω* = 0.86, in the current sample).

Well-being was assessed using the Warwick Edinburgh Mental Wellbeing Scale (WEMWBS; Tennant et al., 2007). The WEMWBS is a 7-item measure designed to assess levels of mental well-being over the preceding 2 weeks (e.g., “I’ve been feeling relaxed”) and was included in the study in case the sample had such low levels of depression, anxiety, and stress that there would be a floor effect. Items are rated on a 5-point Likert-type scale (from 1 = “None of the time” to 5 = “All of the time”). The scale had adequate internal reliability (T0: Cronbach’s *α* = 0.78, McDonald’s *ω* = 0.77; T1: Cronbach’s *α* = 0.80, McDonald’s *ω* = 0.79, in the current sample).

Different components of mindfulness were assessed using the 15-item Five Facet Mindfulness Questionnaire (FFMQ-15; Baer et al., 2008). The FFMQ-15 is a 15-item short form version of the 39-item Five Facet Mindfulness Questionnaire (FFMQ; Baer et al., 2006). The questionnaire contains five sub-categories: non-reactivity, observe, acting with awareness, describe, and non-judging. Items are rated on a 5-point Likert-type scale (from 1 = “Never or very rarely true” to 5 = “Very often or always true”). As this is predominantly a sample of meditators, in accordance with Gu et al. (2016), subscales are calculated, using the original scoring method as described by Baer et al. (2008), as the sum of the three items for each of the five subscales. Overall mindfulness is calculated using 4 of the subscales: non-reactivity, acting with awareness, describe, and non-judging (omitting observing). Participants are asked to respond to “what is generally true for you” (e.g., “When I have distressing thoughts or images I am able just to notice them without reacting”). The scale had good internal reliability (T0: Cronbach’s *α* = 0.90, McDonald’s *ω* = 0.89; T1: Cronbach’s *α* = 0.84, McDonald’s *ω* = 0.83, in the current sample).

Post-course, participants completed the same pre-course measures again, and, in addition, completed questions about unpleasant experiences and harm developed by Baer et al. (2021). These questions were designed to explore any unpleasant experiences, perceived harm, and any support participants received during the course. The response options for the questions were a mixture of Likert-type scales and free-response. Before the questions on unpleasant experiences, a statement about the nature of unpleasant experiences and sensations that participants might have experienced during the course was given and participants responded as to how often during the course they experienced feelings and sensations like the ones described in the statement (from “Never” to “Daily or almost daily”) and how upsetting those experiences were (from “Not at all” to “Extremely”—the exact wording of the question is given in Supplementary Information 1, Table 3). Participants could also answer a free-response question to allow them to elaborate on any unpleasant experiences they reported. Participants then reported any harm they experienced during the course: “By harm, we mean were you worse off in any way, after the course than you would have been if you hadn’t done the course” and reported on how harmful the course was to them (from “Not at all” to “Extremely”) and gave a free-text response to elaborate on any harm they experienced. Participants were then asked about any support they received during the course, who they approached, whether the support was adequate, and if they did not seek support, why they chose not to do so. They were able to report on three instances of support they received. Participants were also asked if they had any other comments about the course.

### Data Analyses

Means and standard deviations were computed for symptom measures (measuring anxiety, depression, and perceived stress; PHQ-9, GAD-7, and PSS, respectively), well-being (WEMWBS), and mindfulness (FFMQ-15). Mirroring the Baer et al. (2021) paper, reliable change was calculated using the algorithm developed by Evans et al. (1998), who define “reliable change” as a function of the baseline SD of the measure and its reliability (using Cronbach’s *α* scores). Using this algorithm, reliable change for the PHQ-9 (depression) was calculated as a change in raw score of 3.45 or more (in either direction); reliable change for the GAD-7 (anxiety) was calculated as a change in raw score of 2.71 or more (in either direction). In accordance with the Baer et al. (2021) paper, we used the same 5-item categorization, using reliable change and the original measures’ cutoffs for clinical significance: (a) “reliable and clinical improvement,” (b) “reliable improvement,” (c) “no reliable change,” (d) “reliable deterioration,” and (e) “reliable and clinical deterioration.”

## Results

Courses ran between January and December 2020. Of the nine courses, one met in person prior to the pandemic, a second was on-going as restrictions were imposed and moved to on-line, and the other seven were during lock-down and all sessions took place on-line. It took an average of 50 days (just over 7 weeks) to complete the course (M = 50.11, SD = 3.16). Attendance was high, as teachers offered individual catch-up sessions where possible for those who missed a session. Completion of four or more sessions is the commonly used definition of whether participants have received an “adequate” experience of mindfulness to provide valid outcome data. Four participants dropped out before four sessions were completed and another dropped out after four sessions. The mean number of sessions attended for the remainder was 7.7 (SD = 0.68). The pre-course questionnaire was completed an average of 1 day before the course started (M = 0.67, Mdn = 1.00, SD = 8.30). The post-questionnaire was completed an average of 16.83 days after the course finished (M = 17.38, Mdn = 5.50, SD = 47.28).

### Reliable Change

Mean levels of depression (PHQ-9), anxiety (GAD-7), and perceived stress (PSS) all significantly decreased pre- to post-course (with Cohen’s *d* effect sizes of 0.56, 0.53, and 0.36, respectively—see Table 2. The recommended cutoffs for Cohen’s *d* are as follows: above *d* = 0.2 is considered a “small” effect size; above *d* = 0.5 is considered a “medium” effect size; above *d* = 0.8 is considered a “large” effect size). Mean levels of mindfulness (FFMQ) and well-being (WEMWBS) both significantly increased (with Cohen’s *d* effect sizes of 0.65 and 0.54, respectively; see Table 2).

**Table 2:** Pre- and post-course scoring for the main outcomes (PHQ-9, GAD-7, PSS, WEMWBS, and FFMQ-15)

Outcome	Pre-course training scores (T0)		Post-course training scores (T1)		Differences		Effect size	Reliable change (T0–T1)				
	Mean (range)	SD	Mean (range)	SD	*t* (df)	*p*	Cohen’s *d*	Reliable and clinical improvement % (*n*)	Reliable improvement % (*n*)	No reliable change % (*n*)	Reliable deterioration % (*n*)	Reliable and clinical deterioration % (*n*)
PHQ-9**	6.22 (0.0–21.0)	4.36	3.85 (0.0–11.0)	2.72	4.30 (58)	.000*	0.56	1.7 (1)	35.6 (21)	57.6 (34)	5.1 (3)	0 (0)
GAD-7***	6.05 (0.0–21.0)	4.61	3.88 (0.0–16.0)	3.09	4.09 (58)	.000*	0.53	5.1 (3)	33.9 (20)	54.2 (32)	6.8 (4)	0 (0)
PSS	15.63 (3.0–30.0)	5.74	13.51 (2.0–28.0)	5.80	2.77 (58)	.008*	0.36					
WEMWBS	22.25 (16.9–35.0)	3.14	24.11 (16.9–35.0)	2.85	− 4.15 (58)	.000*	0.54					
FFMQ-15	52.66 (28.0–72.0)	9.14	56.50 (39.0–72.0)	7.82	− 4.95 (57)	.000*	0.65					

^*^p < 0.01

The sample had low levels of clinical depression and anxiety at baseline, with only four participants (6% of the 72 participants who completed baseline measures) over the clinical threshold for anxiety and one of these four (1%) also over the threshold for clinical levels of depression. At post-course, all but one of these participants dropped below clinical significance for both depression and anxiety. The one participant (with clinical levels of anxiety), who did not fall below clinical significance post-course, nevertheless reported a small reduction in their anxiety symptoms. No participants crossed the threshold from non-clinical levels of anxiety or depression pre-course, to clinical levels post-course. For depressive symptoms, 2% of participants showed reliable and clinical improvement (*n* = 1), 36% showed reliable improvement (*n* = 21), 58% showed no reliable change (*n* = 34), 5% showed reliable deterioration (*n* = 3), and no participants showed reliable and clinical deterioration. For symptoms of anxiety, 5% of participants showed reliable and clinical improvement (*n* = 3), 34% showed reliable improvement (*n* = 20), 54% showed no reliable change (*n* = 32), 7% showed reliable deterioration (*n* = 4), and no participants showed reliable and clinical deterioration (see Table 2 for full details). There were five participants overall who showed reliable deterioration in either depression or anxiety (and two of these participants reported reliable deterioration in both).

### Unpleasant Experiences/Harm

#### Difficult and Unpleasant Experiences

Participants were asked how often the course led them to have “unpleasant thoughts, feelings and sensations such as agitation, sleepiness, upset, uncertainty, etc.” Of the 69 participants who responded, 25% responded “Never” (*n* = 17), 26% responded “Occasionally” (*n* = 18), 10% responded “Less than once a week, but several times during the course” (*n* = 7), 12% responded “About once a week” (*n* = 8), 15% responded “Several times a week” (*n* = 10), and 13% responded, “Daily or almost daily” (*n* = 9). Of the five participants who reported reliable deterioration in depressive and anxiety symptoms, two responded “never,” one responded “occasionally,” one responded “Several times a week,” and one responded, “Daily or almost daily.”

Forty-two participants who reported experiencing “… unpleasant thoughts, feelings and sensations such as agitation, sleepiness, upset, uncertainty” more frequently than “Never” were given the opportunity to respond to a question about how upsetting they found these experiences. Seven participants responded. Two of the seven responded, “Not at all,” and five responded “Somewhat” (*n* = 5), with no participants responding “Quite a bit” or “Extremely.” Six participants provided qualitative information about these unpleasant experiences; participants described cognitive elements (self-judgment, negative feelings, and “unpleasantness at the back-of-my-mind”) and physical elements (heart beating fast, restlessness; anonymized responses are listed in full in Table 3, Supplementary Information 1). Of the five participants who reported reliable deterioration in depressive and anxiety symptoms, only one responded to this question about upsetting experiences, stating that they found these experiences “somewhat upsetting,” and they described these experiences as “unpleasant flashbacks from the past.”

#### Harm

Sixty-nine participants responded to the question “How harmful was the course to you?” The question clarified that “By harm, we mean: were you worse off in any way, after the course, than you would have been if you hadn’t done the course.” All participants (*n* = 69) responded “Not at all”; correspondingly no participants responded “somewhat,” “quite a bit,” or “extremely.” As no participants reported any harm, they were not asked follow-up questions about the harm they had experienced or if they sought any help or support.

#### Differences Between Novice, Intermediate, and Advanced Mindfulness Experience at Pre- and Post-interventions

To explore differences between levels of mindfulness practice experience, we created three experience levels: novice (under 12 months practicing mindfulness, including no experience at all, *n* = 13); intermediate (1–5 years’ experience, *n* = 32); and advanced (over 5 years’ experience, *n* = 27). There were only significant differences found between groups at baseline: for perceived stress (PSS) *F*_(2,69)_ = 3.14, *p* = 0.05; well-being (WEMWBS) *F*_(2,69)_ = 5.53, *p* = 0.006; and mindfulness (FFMQ-15) *F*_(2,69)_ = 4.60, *p* = 0.013 (for means (SDs), see Table 6, Supplementary Information 4). Descriptive statistics showed that those participants who had more than 5 years practicing mindfulness (advanced) had higher levels of mindfulness at baseline than those who had practiced for 1–5 years (intermediate) or for under a year (novice). Novices reported higher levels of mindfulness at baseline than those with an intermediate level of experience. Novice and intermediate participants had similar levels of stress at baseline, and both reported more stress at baseline than advanced participants. However, there were no significant differences between groups post-course.

#### General Post-course Comments

Participants were also given the opportunity to give general feedback about the course (see Supplementary Information 3 for full anonymized responses). Thirty-eight participants responded. There were some mentions of difficult experiences, such as “I noticed an increase in anxiety during the course (or an increase in noticing anxiety). This caused some distress but I think it’s good to be aware,” “I only had one occasion of ‘upset’ which was in the wording used to describe what meditation is,” and “To be honest, it was a difficult experience, and turning towards difficulty, I put my ‘depression’ on the workbench of the mind. On the other hand, letting the tears and angst out was cathartic. At the end, I felt better about life and better [equipped] to face challenges.” However, the course responses were overwhelmingly positive, such as “Thank you for the subtle yet highly effective way in [*which*] I was enriched, sustained and deepened by the practices,” “I am happy to have taken part!,” and “The course was deeply nourishing.”

Of the five participants who reported reliable deterioration in depressive and anxiety symptoms, four responded with comments on the course (see Table 4—Supplementary Information 1 for details). Participants acknowledged that there were challenges either with the course, “I found the course challenging and confrontational,” or with their personal circumstances, “During the course I had some friendship—relationship difficulties as well as an accident injuring my leg which both caused me pain and meant I couldn't exercise which is an important part of my life,” but all four participants also described positive aspects or benefits of the course, for example, “The course has been deeply beneficial to me” and “I think the course is great, the best [I’ve] done.”

## Discussion

The aim of the current study was to investigate whether a follow-on program that offers specific practices to increase awareness of “feeling tone” (*vedanā*) could be effective in reducing emotional distress (depression, anxiety, stress) and improving well-being and mindfulness in those who have previously attended some form of mindfulness training. Furthermore, we aimed to investigate the frequency and severity of unpleasant experiences and harm, and whether any benefit or harm was related to the amount of prior experience of mindfulness practice.

Despite the low levels of psychological ill health in the sample, participants reported significant improvements pre- to post-course on stress, anxiety, depressive symptoms, well-being, and mindfulness. This is particularly interesting given that there is very little research on the benefits of further mindfulness training for experienced meditators. Of the five participants who had clinical levels of anxiety and depression at baseline, four dropped to below clinical levels at follow-up (and the remaining participant showed reduced symptoms). When examining reliable changes in depression and anxiety, almost 40% showed reliable improvement in both depressive and anxiety symptoms. Although these proportions may seem small, this was not a clinical or help-seeking sample, so the level of reliable improvement is not expected to be large. To benchmark this, we can compare the current study’s findings with a similar study of schoolteachers and students (Baer et al., 2021). They found 28% showed reliable improvement in anxiety (compared to 39% in this study) and 13% reliable improvement in depression (compared to 38% in this study). Furthermore, finding relatively modest improvement in samples who are healthy and not seeking help does not imply that the program would not be effective as a standalone program in clinical settings, and further research would be needed to evaluate this.

It is encouraging that no participants crossed the threshold from non-clinical levels of anxiety or depression pre-course to clinical levels post-course. Such instances of reported deterioration might have been expected given the reported rates of adverse outcomes in the meditation and general psychotherapy literature (Baer et al., 2019; Lambert, 2013). Although without an active control condition we cannot attribute this outcome to the program, these findings are encouraging, especially given that the courses took place during the onset and spread of the COVID-19 pandemic where globally people were experiencing increased psychological ill health (W. Cullen et al., 2020).

When exploring differences between levels of meditation experience (novice, intermediate, advanced), there were only significant differences found between groups at baseline for well-being and mindfulness, with the expert meditators showing higher levels of both well-being and mindfulness. Interpreting these differences is limited by not knowing precisely the type, duration, and frequency of ongoing meditation practice in the participants, and since differences in these variables might affect self-rated mindfulness, these on-going practice details should be included in future research. Interestingly, no significant differences were found between groups post-course. It may be the case that those participants with a great deal of mindfulness experience may reap fewer benefits compared with those who are newer to mindfulness, but it is encouraging to note that even the advanced meditators reported improvements, and that those new to mindfulness practice also found that the program benefitted them.

Unpleasant experiences and harm were assessed in the current study using the methods outlined by Baer et al. (2021). We found three-quarters of participants reported difficult or unpleasant experiences such as “agitation, sleepiness, upset, [or] uncertainty.” Given that this sample is composed mostly of participants who have prior experience of mindfulness, it is interesting to note that they reported slightly more unpleasant experiences than the novice samples of students and schoolteachers in Baer et al. (2021), where two-thirds reported unpleasant experiences. Yet, these additional reports of unpleasantness are not surprising given that the aim of the program is to increase awareness of the valence of all experience. Taken as a whole, the reports by participants suggest that difficulties remained at a manageable level. It could also be the case that the experienced meditators expect to turn towards difficulty in these courses and see it as an opportunity to practice these skills.

Although only five participants reported reliable deterioration in depression and anxiety in the current study, it is interesting to note that from the descriptive statistics there seemed to be no relationship between reporting unpleasant experiences and reliable deterioration. Two of these five participants reported no unpleasant experiences at all, and the remaining three reported varying levels of difficulty. It may be the case that the deterioration was unrelated to their course experience, but rather the result of external factors (the same could also be said of reliable improvement). It is also possible that ratings of deterioration might have been due to a response bias created by increased experiential awareness, rather than actual deterioration, though it is important not to dismiss reports of deterioration. Including “post-then-pre” assessment of the same measures (where participants are also asked at post-treatment to re-rate their pre-treatment measures) might be used in the future to clarify this. Of the forty-two participants who reported difficult or unpleasant experiences, it is noteworthy that only seven chose to respond to a question about how upsetting these experiences were; responses varied from “not at all” to “extremely.” We cannot draw any conclusions about why so many chose not to answer this question; possible reasons could be lack of interest, questionnaire fatigue, error, or avoidance. Only six participants gave a qualitative response about their unpleasant experiences; their comments highlighted that they had experienced a range of cognitive and physical sensations that were difficult or unpleasant for them. But all six participants reported that they perceived these experiences to be “not at all” harmful. None of these six participants provided general course comments so there were no further insights into their perceptions of the course.

Of the sixty-nine participants who completed the follow-up questionnaire, none reported that they perceived the course to be harmful (that is, *all* participants responded “not at all” to the harm question). This is encouraging, given that the sample included a range of novice, intermediate, and expert meditators. Furthermore, those who experienced reliable deterioration also perceived the course to be “not at all” harmful. It could be the case that they just are unaware that the course is causing them harm, but their general course comments (from four of the five participants) suggest that they did experience difficulty because of the practices or from personal circumstances, but that they also perceived the course to be beneficial. Teachers commented that the trauma-sensitive skills taught in Week 1 were important in allowing participants to handle these situations wisely and with kindness. The other participants’ course comments—listed in full in Supplementary Information 3—were overwhelmingly positive and highlight that many of the participants were experiencing situations in their lives which may have affected their responses, which were unrelated to the course.

This study did not set out to compare this program (with its focus on feeling tone) with existing programs which focus more on *vedanā* as interoception and related hedonic tone (Cayoun, 2011), nor on the possible mechanisms of change. The question arises whether it will be possible to distinguish such subtle differences in outcome or mechanism at the current state of knowledge, especially since the most widely used secular programs emphasize interoception and the current course builds on these practices. Kerr et al. (2013) point out that it remains unclear whether the “body sensation” element explored in MBPs makes a *unique* contribution to the mechanisms that underlie the changes. This applies to the current program that teaches participants to explore feeling tone explicitly and those like MBSR/MBCT that teach it implicitly, as well as other programs such as MiCBT that focus on *vedanā* as body sensations. Many mechanisms have been nominated in the literature as critical mediators of change, and meta-analyses conclude that there are several plausible processes that co-exist and may share variance (Alsubaie et al., 2107). The benefit of co-emergence theory such as Cayoun and Shires (2020) is that it picks up a vital theme of the Buddhist approach to consciousness—co-dependent arising, a theme also of those theories pointing to changes in whole *modes* of mind (Segal et al., 2013, chapter 4; Williams, 2008). In both traditional and current psychological theories, different elements emerge or arise together, each making its own contribution to the gestalt, so that bringing about change in any one element will affect the whole, to either escalate or reduce distress.

### Limitations and Future Research

There are several limitations in this study. First, this was a preliminary open trial so it cannot assess the size of effect relative to no-treatment or against other active follow-on approaches that might have been used: the effects of this new course might have been brought about by *any* sort of follow-on course that encouraged participants to re-engage or intensify their practice, so we cannot attribute these effects to the specific practices taught.

Second, we did not assess the amount of practice that participants carried out during the week. Objective measurement of this would have required using technology that was not available for this study. Although some studies find a correlation between amount of formal practice and outcome (e.g., Crane, et al., 2014), when assessing harm it is hard to make any inference from such a correlational measure. Someone who is finding practice difficult or traumatic might do less practice, and the data would show a spurious correlation between reduced amount of practice and increased harm. In due course, there needs to be a randomized controlled trial which independently varies the amount of practice per day. Until there is such a trial, it will not be clear whether the amount of practice affects outcome—either beneficial or harmful. And it is important to take account of the possibility that for those who have already completed a mindfulness course, “safety” in relation to practice might mean something different from its meaning for novices. Thus, previous literature on adverse reactions might have limited relevance for a follow-on study such as this. However, the results found no relation between prior experience of mindfulness practice and reports of difficult experiences: those with little or no previous experience did not report more adverse reactions.

Third, because the study recruited participants from those who had completed previous mindfulness courses, the sample might have been biased towards those who found mindfulness of benefit in the past, so it cannot be concluded that this program would have equivalent effects if offered to a sample that consists of “treatment non-responders.” On the other hand, if the sample was biased towards those who had found mindfulness practice helped them in the past, the observed effect size might have been limited by a floor effect, with much of the potential benefit having already accrued. A specific trial for treatment non-responders to examine this issue is needed. Neither could the study assess whether the *type* of previous experience had a bearing on the response to this program. It is possible, for example, that those who had particular *vipassana* retreat experience may have responded differently, and further research is needed on whether and for whom a “match” or “mismatch” between prior mindfulness practice and follow-on programs is beneficial. It was not ideal to have such heterogeneity within the current sample and future research needs a study in which this aspect is controlled as part of the design.

Fourth, future research would benefit from including measures that would help determine factors that would affect the amount of benefit of any program that aims to explore feeling tone. Particularly useful would be measures of alexithymia (which might limit skill development) and interoceptive sensitivity (Dunn, et al., 2010; Farb, et al., 2015) or valence focus (Barrett, 2006) which might help skill development.

A final limitation is that the study was not set up to formally assess feasibility as defined in the framework developed by Bowen et al. (2009) who list eight dimensions along which feasibility of a new program might be evaluated. Despite this, it appears that the study has at least partially been able to address five of these. The *Acceptability* of the program was confirmed from the free responses and by the fact that although participants experienced unpleasant experiences (as we had expected) no one reported that this was harmful. The *Implementability* of the program was evidenced by teachers welcoming the ease with which having the complete set of detailed teaching materials available to them, session by session, helped them to teach it. *Practicality* was evidenced by the data showing program attendance was very high. *Integration* was evidenced by the extent to which teachers could readily incorporate the program within existing teaching schedules and also how participants themselves integrated their new skills in everyday life, as recorded in their free responses. *Preliminary effectiveness* was established in the effect sizes and indices of reliable change in the outcome measures and benchmarking these changes against other studies of established programs with similar populations. Future studies should examine feasibility formally, considering as well other dimensions we did not address: *Demand* (gathering data on use of a new program), *Expansion* (success of the program for a different population or setting), or *Adaptation* (changing the program to adapt to new situations).

The original aim was to develop a program for those who had already completed a course, but some of the teachers who taught the new program suggested in their feedback that it might be an alternative “first taste” of mindfulness, as it (a) takes account of recent developments in trauma-sensitive practices (Treleaven, 2018), (b) offers a variety of types and lengths of practice, and (c) is embedded in the most recent psychological science (e.g., Barrett, 2018; Clark, 2015). It was reassuring that the study found that relative newcomers to mindfulness meditation found the course beneficial and showed no adverse effects. Although this remains possible, the evidence base for MBSR and MBCT is now so extensive that we do not recommend that this program is used as a substitute. Nevertheless, an alternative approach to what could be perceived as a program to “patch” gaps in these existing programs could be to modify MBSR and MBCT in a way that includes practices that explore feeling tone, as defined in this program. For now, however, what this program aims to provide is a follow-on for those who wish to deepen practice by exploring gateways into practice they have not explored before.

In summary, the study provides preliminary indications that this program focusing on feeling tone is effective and safe, resulting in lower levels of stress, anxiety, and depressive symptoms, and higher levels of well-being and mindfulness, with low levels of deterioration, pre- to post-course. It is suggested that this course is used to complement the well-established routes of MBCT and MBSR.

## Supplementary Information

Below is the link to the electronic supplementary material.

Supplementary file1 (PDF 113 KB)

Supplementary file2 (PDF 16 KB)

Supplementary file3 (PDF 124 KB)

Supplementary file4 (PDF 58 KB)

## Data Availability

All de-identified data are available at the Open Science Framework (https://doi.org/10.17605/OSF.IO/EZWND).
