# Supplementary material for: What Next After MBSR/MBCT? An Open Trial of an 8-Week Follow-on Program Exploring Mindfulness of Feeling Tone (vedanā)
Source: Mindfulness (N Y). 2022 Jul 7;13(8):1931–44. doi: 10.1007/s12671-022-01929-0 (PMC9261229; doi:10.1007/s12671-022-01929-0)
Supplement: Supplementary file 1 — Supplementary file1 (PDF 113 KB) [file 12671_2022_1929_MOESM1_ESM.pdf]

Supplementary Information 1: Free Text Responses About Unpleasant Experience and Reliable Deterioration

Table 3:

Free-Text Responses About Unpleasant Experience

Participants were given the statement *‘The practice of mindfulness involves becoming aware of the full range of our experience, pleasant, unpleasant and neutral. This can be challenging, as it sometimes puts us in touch with difficult thoughts and feelings. Learning to manage these thoughts and feelings is part of the mindfulness course and takes time. While it is going on, people doing a mindfulness course can experience unpleasant thoughts, feelings and sensations such as agitation, sleepiness, upset, uncertainty, etc.’* and were asked *‘How often did the mindfulness course lead to you having experiences like the ones described above?’* and then given the chance for free responses (see below).

Unpleasant Thoughts / Feelings	During weeks two and three there was quite a lot of self-judgement about noticing pleasant and unpleasant. "Am I allowed to feel this? " "I shouldn't be experiencing this as unpleasant, most people would be grateful for this experience"
	I could name the unpleasantness that has been at the "back of my mind". Once I had named it I could manage it much better.
	I had two themes or situations I needed to confront others (which is tough for me normally). Once I opened up to feeling the negative feelings they came through quite strongly in my meditations and I felt for a couple of weeks these "preference" or "judgements" of negative feelings came in quite strong but then I realised how powerful it was to feel them and to have compassion about not "holding" onto this energy and not having to believe them (in a weird way). I still felt the negative feelings but slowly became ok with them and this became very apparent in my life outside of meditation where I [found] myself not having to convince others of my perspective.... its ok to not agree... this is a BIG shift and I believe has a lot to do with the course, the material and process and [mindfulness instructor name] and the group she has assembled. Love it
	Unpleasant flashbacks from the past.
Unpleasant Feelings / Physical sensations	Feelings of anxiety, heart beating fast, restlessness

Unpleasant Physical Sensations	*When calling to mind difficulties for the purposes of meditation, the experience felt very safe, as it was always in the context of understanding and compassion. Occasionally, the challenge was not to run away with the thoughts that accompanied them. There were no sensations that occurred because of the meditation only; they were things happening to me already that I brought to mind for the purposes of meditation. Body-focused meditations also allowed me to access some physical discomfort I was experiencing due to an illness in a very accepting way.

*Participant responded ‘Not at all’ to the question ‘How upsetting were these experiences. All other responses were from participants who had responded ‘Somewhat’.

Table 4

Free-Text Course Comment Responses from those Participants Showing Reliable Deterioration

Difficult Course Experience	I found the course challenging and confrontational, but at the same time deeply nourishing and kind. It took me to very deep layers within myself - but in a very grounded, held manner. I never felt out of my depth. It has given me new set of tools that will help me navigate life in a more skillful manner. I am deeply grateful to have been on this course. It has been life changing.
Difficult Personal Experiences	I had a work and personal difficult experiences that was building up over a few months and culminated in the last month of the course. The course leader [mindfulness teacher] held me with such kindness and compassion. It enabled me to surrender and control my respond to the situation.
	During the course I had some friendship - relationship difficulties as well as an accident injuring my leg which both caused me pain and meant I couldn't [exercise] which is an important part of my life. So I found I was unable to give the course the attention it needed. I didn't feel I did it justice. Things that could have helped me didn't so much as I slipped back to negative thinking very easily. In saying that I now understand grounding and can use breath, feet, hands to do that. I now meditate each day and that is a normal part of my life. I find the audios very helpful and am currently using the befriending self one. Becoming more aware of my feelings and thoughts is ultimately helpful and knowing they are not necessarily real or right is helpful.

	There is a wealth of info in this course and I will be repeating it. There are things that bring about good feelings like ten finger gratitude.<br>I think the course is great, the best I've done.
Exclusively Positive Experience	Thanks. I am so grateful for this course. I'm noticing myself acting with calm and finding my breath without trying more each week. The course has been deeply beneficial to me.
