# Supplementary material for: What Next After MBSR/MBCT? An Open Trial of an 8-Week Follow-on Program Exploring Mindfulness of Feeling Tone (vedanā)
Source: Mindfulness (N Y). 2022 Jul 7;13(8):1931–44. doi: 10.1007/s12671-022-01929-0 (PMC9261229; doi:10.1007/s12671-022-01929-0)
Supplement: Supplementary file 2 — Supplementary file2 (PDF 16 KB) [file 12671_2022_1929_MOESM2_ESM.pdf]

Supplementary Information 2:

Table 5

Differences Between Those who Provided Complete Data and Those who Completed Only the Pre-Intervention Questions, or Only the Post-Intervention Questions on the Main Outcome Measures

Measure	Sum of squares	*df*	Mean square	*F*	*p*
Pre course PHQ9	12.12	1	12.12	.67	.416
Post course PHQ9	29.67	1	29.67	3.54	.064
Pre course GAD7	13.55	1	13.55	.69	.409
Post course GAD7	6.63	1	6.63	.65	.423
Pre course PSS	32.90	1	32.90	1.0	.321
Post course PSS	96.06	1	96.06	3.12	.082
Pre course WEMWBS	.61	1	.61	.07	.796
Post course WEMWBS	1.70	1	1.7	.18	.670
Pre course FFMQ	.38	1	.38	.00	.951
Post course FFMQ	32.11	1	32.11	.51	.477
