# Supplementary material for: What Next After MBSR/MBCT? An Open Trial of an 8-Week Follow-on Program Exploring Mindfulness of Feeling Tone (vedanā)
Source: Mindfulness (N Y). 2022 Jul 7;13(8):1931–44. doi: 10.1007/s12671-022-01929-0 (PMC9261229; doi:10.1007/s12671-022-01929-0)
Supplement: Supplementary file 3 — Supplementary file3 (PDF 124 KB) [file 12671_2022_1929_MOESM3_ESM.pdf]

Supplementary Information 3:
Free-Text Responses to Course Evaluation Question

A big thank you from the bottom of my heart to [mindfulness teacher]. For all support time amazing Teacher ! Very grateful beautiful poetry and always able to listen find explanation. I am so grateful I was able to learn so many things . Thank you so much to everyone for this course ! Would love to do some retreat! Stay all well and healthy enjoy the sunshine! Namaste
Mindfulness is somewhat new to me but has become a very important aspect in my life and something that I try to share with those around me. I really enjoyed this course but found it challenging as it highlighted aspects of my life where I realised negative habits are holding me back and that as much as I preach self care I don't practice it enough. However, with the tools learnt at mindfulness, I am able to have self compassion and move forward with continual positive reinforcements.
I am happy to have taken part!
Thank you for a well-facilitated and beautifully-structured experience.
I found this course extremely interesting, testing and had a sense of an adventure. I marvel at how my mind never stops and enjoyed the kindness and friendly curiosity approach of this course.<br>Thank you.
I really enjoyed the program and sharing with the group. I did not expect anything and feel that I grew. I now know myself better. I think that the combination Yoga + MBSR + private therapy session is the best way for me to grow and take care of myself as body and mind. I came across yoga and MBSR thanks to my personal therapist.<br>Thanks to the course, I now have plenty of tools to be more mindful and aware of my thoughts and feelings. I will keep practicing MBSR. I feel that being in the group during the course helped me practicing. I will try to reach out some people from the group and suggest to keep practicing once a week together.<br>I am at the start of my journey and I feel that I have a lot to learn about Mindfulness Practices and myself.
[Mindfulness teacher]'s guidance was a blessing, she really managed to create a safe and kind virtual space.<br>It was a real enrichment to have a real diversity of profile among the participants (age, profession, experience of meditation...).<br>The material and invitation shared at the end of each session supported a very rich and beneficial self-practice for the rest of the week.<br>Also very grateful to [mindfulness teacher] for being so available. Just knowing she was here if we needed support in-between the sessions was very supportive.
This process of mindfulness practice has helped me to develop a compassionate heart for me. Most useful in this discovery was the themed weeks that addressed: Finding ground, feeling tone (good, bad and neutral, and befriending the mind). Earlier discoveries from the MBSR helped me focus attention, respond differently using an attitude of: non-judgment, loving and kindness, patience, gratitude, compassion and humbleness. However, I discovered that in practicing these efforts, I focused attention on the relational space and in so doing, forgot about me. This course has helped me identify the neglected me.

Previously, the focus of my attention was on the relational space between me and others. From the perspective of my work, it benefited the client-therapist relationship; and, in my personal life it benefited the relational space. One of the most difficult discoveries was my developing awareness of personal neglect. My previous efforts were not in vain, but, they contributed to maintaining an unbalanced relationship whereby my enduring efforts at my relational space resulted in self-neglect.<br><br>Going forward, there is a transformation. I am paying more attention to the reality of my situation with an open and honest attitude that gives cognizance to my feelings and interpretations of how things are. I have always struggled to trust my judgement because of feelings of self-doubt. The reference to grounding helps me to stand firm in what I know and experience (sounds like a reality check). Decision making going forward will be difficult as it involves a process whereby I will be standing my ground, centering, and maintaining my equilibrium as opposed to the equilibrium of the system.<br><br>The equilibrium that I refer to is no longer about maintaining the status quo, instead it refers to addressing a problem (a truthful problem) in order that I can find inner peace (albeit transient). I suppose it really refers to befriending me with a forgiving, compassionate heart.
VERY GOOD
I found the course helpful in extending my practice - bringing in the different anchor points consistently, taking a slow and gentle route back to the body once the mind wandered, forming a nest of befriending self before turning towards the difficult.<br><br>I was aware that cultivating kindness towards myself sometimes had an edge to it in that I just let myself become take a lazy route, or a self indulgent route. This shadow of kindness I think is common. Of course, it is ultimately unkind to move not practice or to let oneself consistently off the hook. It was an edge I worked with on this course<br><br>Many thanks to you. All gratitude and a deep bow.
Thank you! I appreciate my capacity to now look at a thought , feel into the tone when available and also to put it on the workbench. These tools has added another dimension to my practice, a way to be more fully with my experience. Thank you [mindfulness teachers]! Such a gift. A deep bow of gratitude to you.
Thank you so much [mindfulness teacher], this was beautiful. Leaving this course with an awakened mind, fuller days and a deeper appreciation for the ordinary, which when attended to turned into wildly significant moments and feelings.
I noticed an increase in anxiety during the course (or an increase in noticing anxiety). This caused some distress but I think its good to be aware.
It was helpful to have another structured course to help deepen and expand my mindfulness practice.<br>The one practice I found difficult was the practice of assigning a feeling tone (pleasant/unpleasant) to my thoughts. I found that it was often difficult to decide one way or the other and that the subsequent thought process of trying to decide was distracting.

I found the course extremely useful, deepening my practice for sure. A number of unfortunate events ([family death, separation, family conflict]) took place for me during this course. None were within my control, and they had nothing to do with the course. It was however, very useful to be doing the course whilst experiencing these negative events. The course content helped me to cope better. I was strangely peaceful throughout, despite my circumstances. My concern is that some of the answers I have given in this questionnaire may be reflective of my circumstances, and not be a true reflection of this course and its benefits.
This course has given me the opportunity to soften my protective walls a bit more, which did make me more sensitive and vulnerable in my line of work as well. Through that and an increase in work load I took some strain, but was able to address the situation and put changes in place.<br>I absolutely enjoyed the sense of deepening my practice through the course and also the ability to integrate the mindfulness attitude into my daily life better.<br>the shorter, 10 and 15 min meditations worked well for me and having them downloaded on the phone was a bonus ... always available (waiting between patients, in the car, ...).<br>connecting through my body's sensations ... the going upstream ... helped me "come out of my head and into my heart".<br>[The Mindfulness teachers] were great facilitators and the group very responsive and intuitive.<br>A great course and to be highly recommended:)
The course was deeply nourishing. For me it was the deepening and sustaining experience that I had hoped for. Specifically:-<br>The new anchors of feet, seat and hands I am using often in my daily practice. Especially the seat, which I feel has grounded my practice in a very physical and fundamental way and allowed me to settle more deeply onto my cushion.<br>Attending more closely to the feeling tone in my body at different points in the day felt very kind and caring. Almost like a friend checking in with me. It wasn't always easy, but it felt nourishing.<br>The befriending practice has been so comforting over the past month, with all the uncertainty and anxiety. Just the words - may I be safe and at peace in the midst of this - has been very useful to me.<br>thank you.
I only had one occasion of "upset" which was in the wording used to describe what meditation is. Obviously, I didn't relate to this description and it went against my deep inner experience of meditation. For the rest, the course was incredibly empowering, helpful, nurturing and uplifting. I would do it again in a heartbeat.
What a beautiful experience. [Mindfulness teacher] facilitated it with kindness, wit and insight, and held the group expertly. I loved the simplicity and focus of the course, which really helped to integrate what I learnt.
I found the course more than lived up to my expectations. I developed greater awareness, got deeper into my practice and re-experienced kindness to myself which was beneficial to others around me too.
The course was well led by a very knowledgable, compassionate and committed facilitator who managed the online aspect of the course well. I really appreciated the opportunity to connect with others and would be interested in any follow-up sessions. Thank you.

The practice activates much awareness about emotions and soul sensations. I now let feelings of joy easily. They come in a form of joyous songs that run on and on in my heart.<br>This is a very pleasant practice for it stabilizes me in the event of an unpleasant instance. I am able to detect and defer and continue focused on the pleasant and planned project.<br><br>I am very thankful for the deepening experience I have gained whilst doing the course. I trust to grow it more with time.
Thank you for the subtle yet highly effective way in I was enriched, sustained and deepened by the practices and the community of learners and teachers. We learned and grew together and we also taught each other so much along the way. The course was guided by two very wise and kind beings
I found this course more 'difficult' than the original MBSR that I did. I did not experience the massive shift that I had during the first course I did. I know that perhaps it was an unrealistic expectation, but my first course had such a profound effect on me that I suspect I was slightly disappointed that my experience of this one was somehow 'less'.<br>Also, [family member] passed away during this course and I was flooded with all sorts of emotions that made it harder to immerse myself fully in the experience of the course.<br>Loved the participants though! They were all fully committed 'mindfulness' people that made it a beautiful safe place to be for the duration of the course.
I had a work and personal difficult experiences that was building up over a few months and culminated in the last month of the course. The course leader [mindfulness teacher] held me with such kindness and compassion. It enabled me to surrender and control my respond to the situation.
Although it was delivered via zoom, I especially valued the interaction with others on the course.I felt it was a safe and supportive environment to explore thoughts and feelings
Thank-you. I shared my comments with [mindfulness teachers] during the course.
I found the course extremely beneficial in helping me identify and cope with my anxiety and fear. It gave me a practical way of dealing with my feelings. It taught me to realize the strength of the mind in controlling my thoughts and fears. Becoming more aware of feelings in the mind and body assisted me to feel more in control of my reactions to the anxiety and fears.This is certainly a process and needs to become part of your life through regular meditation and quiet time.I found regular (6 monthly) retreats are critical to assist me to keep on track. The benefits of learning and support from others in the group is huge. (I found this less by doing the course on-line.This course as changed my life for the better--thank you so much.
This course came on top of a load and demands on me that are barely manageable at the best of times. So I did not have great expectations of having loads of time. However, when I tried to practice, in fact I am so depleted that it was more of a struggle than I thought. Almost impossible, in fact. Impossible to be quiet and still. (I had learnt to "cope" by moving, exercising and being extremely efficient).<br>However, that in itself was an awareness that was very much needed and in fact quite healing. During the course the kindness was emphasized. So onwards and upwards but with a deep realization of how much I am in need of care on all levels.
My main aim in doing the course was to find a way to help me to relax my body. I had become very aware of all the tension I was carrying around with me, almost all of the time, in my neck and shoulders, back, arms and hands. I wanted to find a way to be able to systematically relax my body. The course was extremely helpful! Doing the grounding meditation really opened up a way for me to focus on my body - mainly my hands but also me feet at times -

and let go, using my out-breath. I did the meditations just after getting up in the morning and found that relaxing at the beginning of the day gave me a way to start the day calmly and led to less tension building up during the day. It left me feeling deeply grounded. Another meditation at the end of the workday would be ideal to let go of the tension which has built up during the day. I am working on implementing this in my life i.e. making the time for it.

I found that the longer practices - 20 to 30 minutes - worked best for me.

I want to say thank you for saying 'This is meditation'. It really took the pressure off the meditation going any specific way. It was very helpful!

I feel that I am already open to 'see' a fair amount of the beautiful sensory moments in the day: a beautiful cloud, the colour in the sky, sunlight shining through leaves, the smell of a herb, bird song, the taste of hot chocolate, the hoot of an owl, the softness of my scarf. I feel so grateful that I am able to appreciate these things. And I feel more hopeful when I see beauty in the world. When I did the appreciation practices it made me feel joyful, like I was stepping into a beautiful garden.

Things came to a head for me in week 4 of the course when I found myself extremely irritable and close to a 'sense-of-humour-failure' event. The feeling of tension in my body was very acute, and I had a sense of how deep stress runs in me, how intrinsic or systemic it is to me. (I had had a [medical] appointment that day, with a difficult procedure. That may have been part of my general state but it felt like there was more to it than just being under the weather because of the [appointment]) The walking meditation - although I had an instant dislike of it from before and when I read that it was the topic of session 4 - was actually really helpful in calming me down. I found the mantra ' It's ok not to like this', which I repeated at each turn, especially soothing. Eventually my agitation came down to what felt like manageable levels. I was quite amazed by that and decided to review my opinion of the mindful walking. I didn't get to do the practice during the week but I will remember to access it in the future when I feel really agitated, as a way to soothe myself.

I was already used to doing a 'weather pattern' check-in with myself in the morning as I wake up, from all the personal development I have done over the years. I found doing the feeling tone meditations quite difficult, which surprised me. I thought I would find it easy. Instead I found that I could not access the feeling tone of stimuli around me or thoughts or even events and that most things registered as 'neutral'. I had quite an epiphany about that: I realised that I have developed this 'being neutral' as a coping mechanism to stop myself from reacting to things, to control myself, because that way I could not be overwhelmed, because that way I could not say the wrong thing and cause a counter-reaction. This ties into my very firm belief that actions speak louder than words, that actions are symbolic of our true intent. A spontaneous reaction will reveal how I really feel about something and so I am controlling my reactions to not upset anyone, to be pleasing, which is a great theme in my life. I feel sad that I feel it is necessary to control my self-expression but also hopeful: Now that I have become aware of what I am doing I have a chance to change it. I know I feel much better about myself than I did when I set this control system up and I feel I can risk letting myself out. I have already started to find ways to do this more and more. So perhaps becoming aware of the extent of my control now is just the next step in the right direction.

I came to another difficult moment in session 6 - when we did the feedback on the past week - when one of the course participant's feedback resonated very strongly with me: dissociation and disconnection. It ties into the control of my responses I discovered in session 4. I realised that the disconnection allows me to plod on, how I can cope with doing what I am doing, the way my life is. I suddenly became aware of feeling very stuck where I am - in relation to looking after [family member]. It felt acutely that my whole life is about service, all about duty and sacrifice. Questions which came up were: How did I get here? What about me? Do I want it to be like this? The answer was: I am not sure. And then the thought: I keep myself so small where I could be so big and powerful. And another thought: I resent the expectation that I will do this, that I will not live my life but rather take care of hers.
I realised that all the tension in my body is me ignoring and acting against my feelings, again and again, accumulatively. I wrote that one could say I have become a robot. I just overwrite what I am feeling and do what I feel is expected of me, out of duty or to please. I make so much space for others to be themselves. I would like to make at least the same amount of space to be myself, my true self.
It is interesting that I had made a note in my journal around that time, but before this outpouring, that I am in the midst of a profound personal transformation!

During the main part of session 6 we talked about willingness versus capacity. Thinking about capacity is very helpful to me. I could recognize that I am always full of willingness – to my own detriment, I could say – but that I had never really learnt to even question my capacity, never mind respect it. I feel like I have internalised the question of ‘Do I have capacity?’ a little bit and hopefully it will grow into a beautiful strong tree inside me.

We did an exercise where we practiced putting a difficulty on the ‘workbench of our mind’, standing back to examine it. For me the difficulty was wanting another dog after our last and most special dog died last year and my husband saying that he is not ready to have a new dog yet. When I looked at this inside myself I felt a hollow feeling in my chest and a dark cloud in my mind’s eye. My journal entry straight after this account is: my truth – stepping onto holy ground – awareness – sense of great responsibility towards myself & my journey. I feel like I made a breakthrough in how I want to place myself in my own life. ‘My truth’ connects with giving myself space to be myself and checking on my capacity. Feeling my truth is like stepping onto holy ground. It feels like an absolute, a point where I am no longer negotiable, like drawing a line in the sand. By allowing myself to feel it I am taking on the responsibility for myself and my journey.

In the week after this session came another breakthrough in my life in the form of swimming in the tidal pool again after a long time. I had such joy bobbing up and down in the cold water, in the waves, in the sunshine. I think it broke a dam inside me which had been on the brink of overflowing since week 4. In session 7, during the reflection on the past week, I realised that I had connected with my anger – about the [medical appointment] and my tenant – without feeling bad about it. At times I have a very difficult time with anger. I feel very wary of it at the best of times, afraid most other times. For me it connects with shame because of the way it can leave me out of control. But I had allowed myself to be angry! And it had not consumed me. After a while it just became less and went away. I had also allowed myself to do things which I like doing – swimming in the sea, every day – and I had let go of my inner judgement when I went swimming rather than doing something ‘more productive’.

We talked about the 'funnel of exhaustion'. I could see myself in the funnel in the past, giving up on the experiences which enrich me, one by one, just to be more of service, a better daughter, a better worker. I have come to realise and feel deeply that it actually works the other way around: the more nourishing I am to myself the more I can be of service, a better daughter, a better worker. It's a paradox of sorts! So it is the best for everyone if I give myself permission to have fun, to take a rest, to replenish my reserves, to say 'no' when I do not have the capacity. This is such a big realisation for me that it will take me some time to see the whole new landscape it makes possible. I feel an exciting journey has begun!<br><br>In conclusion [mindfulness teacher] asked us some questions in the last session. Here are some of my answers:<br>1. What was your intention for doing this course? I came to get help with relaxing.<br>2. What have you learnt? I have learnt something about allowing myself to say 'no' and 'It's ok not to like this', about willingness vs capacity and about nourishing myself.<br>3. What do you have difficulty with? Relaxing. I control myself very tightly and push myself down into a very small space, until I am just mind, no feeling, because I fear that the dam will burst if I let out even the smallest bit.<br>4. How do you skilfully manage that? By learning and experiencing that it is ok to let myself out, to live myself, that it is ok to be closer to my emotions; By nourishing and nurturing myself; With kindness and compassion; By learning to check in with my capacity; By understanding that I don't need to be better or even good! And once I can get in touch with the feeling tone of things in meditation practice - because I have allowed myself to feel - then there will be less need to be reactive in real life. I can examine the difficulty on the 'workbench of the mind' and hopefully this will create a distance to it and make it easier to let it go. This will allow me to be less afraid of my emotions, less afraid to get out of control, which in turn will enable me to let myself out more and more. And being myself as I truly am will allow me to relax.
To be honest, it was a difficult experience, and turning towards difficulty, I put my 'depression' on the workbench of the mind. On the other hand, letting the tears and angst out was cathartic. At the end, I felt better about life and better equipt to face challenges.
This course was challenging in that I experienced much difficulty in "going upstream to the feeling tone". I almost always felt like I was ascribing a feeling tone after the fact, because I "had to". So I found myself in my head a lot during those sections of the course.<br>However, the everyday mindful practices/exercises all through were very useful and implementable and many things I will continue to do.<br>I was able to establish a good anchor for myself in my body - I rediscovered breathing in a circular motion along the heavenly circle (something I learned on a qigong retreat a few years ago) - it just popped into my mind during one of the sittings and I have been using it fairly successfully ever since. So the invitation to find alternative anchors has been very helpful.<br>Thank you for the opportunity to try new things to deepen my practice and through all these things that we do, cultivate the resilience that we need to deal with our hectic lives and our hectic world.
The course was so valuable. I started practicing mindfulness after someone lent me the Frantic World book. I worked through it over 8 weeks, and could not believe the difference it made. That was 5 years ago. I have since done a 2 year postgraduate program in teaching mindfulness, and run almost to MBI's. I love this course, it deepened and simplified my practice. I shall continue to benefit from what I have learned here and intend to do work through

the course again on my own for another 8 weeks. I am already integrating aspects of what I have learned into my teaching. I am happier, more focused and effective and more positive than I was before the course.
Thanks I am so grateful for this course. I'm noticing myself acting with calm and finding my breath without trying more each week. The course has been deeply beneficial to me.
I found the course challenging and confrontational, but at the same time deeply nourishing and kind. It took me to very deep layers within myself - but in a very grounded, held manner. I never felt out of my depth. It has given me new set of tools that will help me navigate life in a more skillful manner. I am deeply grateful to have been on this course. It has been life changing.
During the course I had some friendship - relationship difficulties as well as an [injury] which both caused me pain and meant I couldn't cycle which is an important part of my life. So I found I was unable to give the course the attention it needed. I didn't feel I did it justice. Things that could have helped me didn't so much as I slipped back to negative thinking very easily. In saying that I now understand grounding and can use breath , feet ,hands to do that.. I now meditate each day and that is a normal part of my life. I find the audios very helpful and am currently using the befriending self one. Becoming more aware of my feelings and thoughts is ultimately helpful and knowing they are not necessarily real or right is helpful. There is a wealth of info in this course and I will be repeating it. There are things that bring about good feelings like ten finger gratitude.<br>I think the course is great, the best Ive done.
Rich body of material. Was going through a very demanding period at work during the course, including some major reorganization of the corporate business. I struggled to find time to devote to the practice, but the 2 hour weekly sessions and practice sessions during the week were positive anchors. The course was very well facilitated by [the mindfulness teachers] and the participants all made very valuable contributions, many registering profoundly with me. In the weeks since the course I have come back to some of the recordings, especially that related to difficult events. I will continue to do so. Thank you.
This was an absolutely incredible course - it has been brilliantly devised so that every week builds on the previous week. I hit a huge amount of resistance during the walking meditation week that continued to the end of the fifth week. I realise now that it was due to the fact that I was working very closely with my inner critic and somehow the kindness and acceptance with which the course was run was too much for me... if that makes any sense. It was no fault with the course, but simply my own reaction. (I did notice in the group feedback that a number of people mentioned that the found the walking meditation difficult which I found quite reassuring, although perhaps it has more to do with it being week 4 than the actual type of meditation?) I then found that the meditations in week six - exploring difficulty - were completely liberating - the feeling of being able to sit beside myself and feel that it was ok to be grumpy or angry or afraid was completely transformational - I have always had an internal script that being angry or not always friendly and always saying yes is bad or wrong or a personal failing, and the allowing that arrived after all the weeks of working with it being ok and accepting whatever arose, suddenly crystalised into this incredible moment and has freed me up in a profound way. It has allowed me to be more upfront and clear with my family and less fearful to confront tricky issues (we have [family] here with us at home during lockdown since March 27, bar three weeks in June when they were [away] for a few days).<br>I have decided to begin the cycle again from week one, and may do so again after the next eight weeks end.<br>I cannot thank you enough for this course - the meditation recordings are also brilliant and [instructor’s] voice is really soothing and helps with the gentle

acceptance.<br>[Mindfulness teachers] held the space in a very grounded and insightful way and worked so beautifully together. I was initially uncertain how it would work with the Zoom format, and it is obviously a poor cousin of a real meeting, but [mindfulness teacher] managed the technology space really well.<br>I do think that this not being my first introduction to meditation helped, and it is good that it is not an introductory course, but I have to say that it is so well structured, that I believe it would be possible for people new to meditation to benefit, it may just be necessary for it to be a bit slower - esp as week six would have been very tricky for me to do if I was a less experienced meditator.<br>Thank you so much for this opportunity.<br>Wishing you well.<br>[name]
Felt it was very helpful during the lockdown period and the presentation of the course was excellent.
