# Supplementary material for: What Next After MBSR/MBCT? An Open Trial of an 8-Week Follow-on Program Exploring Mindfulness of Feeling Tone (vedanā)
Source: Mindfulness (N Y). 2022 Jul 7;13(8):1931–44. doi: 10.1007/s12671-022-01929-0 (PMC9261229; doi:10.1007/s12671-022-01929-0)
Supplement: Supplementary file 4 — Supplementary file4 (PDF 58 KB) [file 12671_2022_1929_MOESM4_ESM.pdf]

Supplementary Information 4:

Differences between novice, intermediate, and advanced mindfulness experience at pre- and post-intervention.

Table 6

Pre-Intervention Differences Between Novice, Intermediate and Advanced Mindfulness Experience Groups

Measure	Group	N	Mean	Std. Deviation	Std. Error	95% Confidence Interval for Mean		Minimum	Maximum
						Lower Bound	Upper Bound		
FFMQ	Novice (Under 12 months)	13	50.92	8.66	2.40	45.69	56.15	32.00	61.00
	Intermediate (1-5 years)	32	49.94	9.09	1.61	46.66	53.22	28.00	66.00
	Advanced (Over 5 years)	27	57.22	10.40	2.00	53.11	61.34	39.00	74.00
	Total	72	52.85	10.01	1.18	50.50	55.20	28.00	74.00
WEMWBS	Novice (Under 12 months)	13	21.57	2.44	0.68	20.10	23.04	16.88	26.02
	Intermediate (1-5 years)	32	21.39	2.50	0.44	20.49	22.29	16.88	28.13
	Advanced (Over 5 years)	27	23.72	3.32	0.64	22.41	25.04	18.59	35.00
	Total	72	22.30	3.00	0.35	21.59	23.00	16.88	35.00
PSS	Novice (Under 12 months)	13	18.31	5.85	1.62	14.77	21.84	11.00	32.00
	Intermediate (1-5 years)	32	16.66	5.89	1.04	14.53	18.78	5.00	30.00
	Advanced (Over 5 years)	27	13.96	5.03	0.97	11.97	15.95	3.00	25.00
	Total	72	15.94	5.74	0.68	14.60	17.29	3.00	32.00
PHQ-9	Novice (Under 12 months)	13	5.31	4.27	1.18	2.73	7.89	0.00	14.00
	Intermediate (1-5 years)	32	7.00	4.52	0.80	5.37	8.63	2.00	21.00
	Advanced (Over 5 years)	27	5.22	3.78	0.73	3.73	6.72	0.00	13.00

Measure	Group	N	Mean	Std. Deviation	Std. Error	95% Confidence Interval for Mean Lower Bound	95% Confidence Interval for Mean Upper Bound	Minimum	Maximum
	Total	72	6.03	4.24	0.50	5.03	7.02	0.00	21.00
GAD-7	Novice (Under 12 months)	13	5.54	4.33	1.20	2.92	8.16	0.00	13.00
	Intermediate (1-5 years)	32	6.09	4.34	0.77	4.53	7.66	1.00	21.00
	Advanced (Over 5 years)	27	5.70	4.69	0.90	3.85	7.56	0.00	18.00
	Total	72	5.85	4.42	0.52	4.81	6.89	0.00	21.00

Table 7

Post-Intervention Differences Between Novice, Intermediate and Advanced Mindfulness Experience Groups

Measure	Group	N	Mean	Std. Deviation	Std. Error	95% Confidence Interval for Mean		Minimum	Maximum
						Lower Bound	Upper Bound		
WEMWBS	Novice (Under 12 months)	8	23.12	2.77	0.98	20.80	25.43	16.88	26.02
	Intermediate (1-5 years)	28	23.64	2.15	0.41	22.80	24.47	18.59	28.13
	Advanced (Over 5 years)	23	25.02	3.44	0.72	23.53	26.51	19.98	35.00
	Total	59	24.11	2.90	0.37	23.36	24.85	16.88	35.00
FFMQ	Novice (Under 12 months)	8	54.38	8.98	3.17	46.87	61.88	39.00	67.00
	Intermediate (1-5 years)	27	54.78	6.19	1.19	52.33	57.23	45.00	71.00
	Advanced (Over 5 years)	23	59.26	8.63	1.80	55.53	62.99	44.00	72.00
	Total	58	56.50	7.82	1.00	54.44	58.56	39.00	72.00
PSS	Novice (Under 12 months)	8	14.63	6.91	2.44	8.85	20.40	9.00	28.00
	Intermediate (1-5 years)	28	14.21	5.43	1.03	12.11	16.32	6.00	27.00
	Advanced (Over 5 years)	23	12.26	5.87	1.22	9.72	14.80	2.00	22.00
	Total	59	13.51	5.79	0.75	12.00	15.02	2.00	28.00

PHQ-9	Novice (Under 12 months)	8	3.38	2.07	0.73	1.65	5.10	2.00	8.00
	Intermediate (1-5 years)	28	4.64	2.53	0.48	3.66	5.62	1.00	11.00
	Advanced (Over 5 years)	23	3.04	2.96	0.62	1.76	4.32	0.00	10.00
	Total	59	3.85	2.72	0.35	3.14	4.56	0.00	11.00
GAD-7	Novice (Under 12 months)	8	3.75	3.45	1.22	0.86	6.64	0.00	9.00
	Intermediate (1-5 years)	28	4.32	3.21	0.61	3.08	5.57	0.00	16.00
	Advanced (Over 5 years)	23	3.39	2.87	0.60	2.15	4.63	0.00	11.00
	Total	59	3.88	3.09	0.40	3.08	4.69	0.00	16.00
